# Prediction of morbidity and mortality after early cholecystectomy for acute calculous cholecystitis: results of the S.P.Ri.M.A.C.C. study

**DOI:** 10.1186/s13017-023-00488-6

**Published:** 2023-03-18

**Authors:** Paola Fugazzola, Lorenzo Cobianchi, Marcello Di Martino, Matteo Tomasoni, Francesca Dal Mas, Fikri M. Abu-Zidan, Vanni Agnoletti, Marco Ceresoli, Federico Coccolini, Salomone Di Saverio, Tommaso Dominioni, Camilla Nikita Farè, Simone Frassini, Giulia Gambini, Ari Leppäniemi, Marcello Maestri, Elena Martín-Pérez, Ernest E. Moore, Valeria Musella, Andrew B. Peitzman, Ángela de la Hoz Rodríguez, Benedetta Sargenti, Massimo Sartelli, Jacopo Viganò, Andrea Anderloni, Walter Biffl, Fausto Catena, Luca Ansaloni, the S.P.Ri.M.A.C.C. Collaborative Group, Goran Augustin, Trpimir Morić, Selmy Awad, Azzah M. Alzahrani, Mohamed Elbahnasawy, Damien Massalou, Belinda De Simone, Zaza Demetrashvili, Athina-Despoina Kimpizi, Dimitrios Schizas, Dimitrios Balalis, Nikolaos Tasis, Maria Papadoliopoulou, Petrakis Georgios, Konstantinos Lasithiotakis, Orestis Ioannidis, Lovenish Bains, Matteo Magnoli, Pasquale Cianci, Nunzia Ivana Conversano, Alessandro Pasculli, Jacopo Andreuccetti, Elisa Arici, Giusto Pignata, Guido A. M. Tiberio, Mauro Podda, Cristina Murru, Massimiliano Veroux, Costanza Distefano, Danilo Centonze, Francesco Favi, Raffaele Bova, Girolamo Convertini, Andrea Balla, Diego Sasia, Giorgio Giraudo, Anania Gabriele, Nicola Tartaglia, Giovanna Pavone, Fabrizio D’Acapito, Nicolò Fabbri, Francesco Ferrara, Stefania Cimbanassi, Luca Ferrario, Stefano Cioffi, Marco Ceresoli, Chiara Fumagalli, Luca Degrate, Maurizio Degiuli, Silvia Sofia, Leo Licari, Mario Improta, Alberto Patriti, Diego Coletta, Luigi Conti, Michele Malerba, Muratore Andrea, Marcello Calabrò, Beatrice De Zolt, Gabriele Bellio, Alessio Giordano, Davide Luppi, Carlo Corbellini, Gianluca Matteo Sampietro, Chiara Marafante, Stefano Rossi, Andrea Mingoli, Pierfrancesco Lapolla, Pierfranco M. Cicerchia, Leandro Siragusa, Michele Grande, Claudio Arcudi, Amedeo Antonelli, Danilo Vinci, Ciro De Martino, Mariano Fortunato Armellino, Enrica Bisogno, Diego Visconti, Mauro Santarelli, Elena Montanari, Alan Biloslavo, Paola Germani, Claudia Zaghi, Naoki Oka, Mohd Azem Fathi, Daniel Ríos-Cruz, Edgard Efren Lozada Hernandez, Ibrahim Umar Garzali, Liliana Duarte, Ionut Negoi, Andrey Litvin, Sharfuddin Chowdhury, Salem M. Alshahrani, Silvia Carbonell-Morote, Juan J. Rubio-Garcia, Claudia Cristina Lopes Moreira, Iñigo Augusto Ponce, Fernando Mendoza-Moreno, Anna Muñoz Campaña, Heura Llaquet Bayo, Andrea Campos Serra, Aitor Landaluce-Olavarria, Mario Serradilla-Martín, Antonio Cano-Paredero, Miguel Ángel Dobón-Rascón, Hytham Hamid, Oussama Baraket, Emre Gonullu, Sezai Leventoglu, Yilmaz Turk, Çağrı Büyükkasap, Ulaş Aday, Yasin Kara, Hamit Ahmet Kabuli, Semra Demirli Atici, Elif Colak, Serge Chooklin, Serhii Chuklin, Federico Ruta, Begoña Estraviz-Mateos, Izaskun Markinez-Gordobil

**Affiliations:** 1 Division of General Surgery, Fondazione IRCCS Policlinico San Matteo, Pavia, Italy; 2 Department of Clinical, Diagnostic and Pediatric Sciences, University of Pavia, Via Alessandro Brambilla, 74, 27100 Pavia, PV Italy; 3 Hepato-Biliary and Liver Transplantation Department, AORN Cardarelli, Napoli, Italy; 4 Department of Management, Ca’ Foscari University of Venice, Venice, Italy; 5 The Research Office, College of Medicine and Health Sciences, United Arab Emirates University, Al-Ain, United Arab Emirates; 6 Intensive Care Unit, Bufalini Hospital, Cesena, Italy; 7 General and Emergency Surgery, School of Medicine and Surgery, Milano-Bicocca University, Monza, Italy; 8 Department of Emergency and Trauma Surgery, Pisa University Hospital, University of Pisa, Pisa, Italy; 9 Department of Surgery, Madonna Del Soccorso Hospital, San Benedetto del Tronto, Italy; 10 Unit of Clinical Epidemiology and Biometry, Fondazione IRCCS Policlinico San Matteo, Pavia, Italy; 11 Abdominal Center, Helsinki University Hospital and University of Helsinki, Helsinki, Finland; 12 Department of General and Digestive Surgery, Hospital Universitario La Princesa, Instituto de Investigación Sanitaria Princesa (IIS-IP), Universidad Autónoma de Madrid (UAM), Madrid, Spain; 13 Denver Health System - Denver Health Medical Center, Denver, USA; 14 Department of Surgery, University of Pittsburgh School of Medicine, UPMC-Presbyterian, Pittsburgh, USA; 15 Department of Surgery, Macerata Hospital, 62100 Macerata, Italy; 16 Gastroenterology and Digestive Endoscopy Unit, Fondazione IRCCS Policlinico San Matteo, Pavia, Italy; 17 Division of Trauma/Acute Care Surgery, Scripps Clinic Medical Group, La Jolla, CA USA; 18 General and Emergency Surgery, Bufalini Hospital, Cesena, Italy

**Keywords:** Cholecystectomy, Acute cholecystitis, Surgical risk, POSSUM

## Abstract

**Background:**

Less invasive alternatives than early cholecystectomy (EC) for acute calculous cholecystitis (ACC) treatment have been spreading in recent years. We still lack a reliable tool to select high-risk patients who could benefit from these alternatives. Our study aimed to prospectively validate the Chole-risk score in predicting postoperative complications in patients undergoing EC for ACC compared with other preoperative risk prediction models.

**Method:**

The S.P.Ri.M.A.C.C. study is a World Society of Emergency Surgery prospective multicenter observational study. From 1st September 2021 to 1st September 2022, 1253 consecutive patients admitted in 79 centers were included. The inclusion criteria were a diagnosis of ACC and to be a candidate for EC. A Cochran-Armitage test of the trend was run to determine whether a linear correlation existed between the Chole-risk score and a complicated postoperative course. To assess the accuracy of the analyzed prediction models—POSSUM Physiological Score (PS), modified Frailty Index, Charlson Comorbidity Index, American Society of Anesthesiologist score (ASA), APACHE II score, and ACC severity grade—receiver operating characteristic (ROC) curves were generated. The area under the ROC curve (AUC) was used to compare the diagnostic abilities.

**Results:**

A 30-day major morbidity of 6.6% and 30-day mortality of 1.1% were found. Chole-risk was validated, but POSSUM PS was the best risk prediction model for a complicated course after EC for ACC (in-hospital mortality: AUC 0.94, *p* < 0.001; 30-day mortality: AUC 0.94, *p* < 0.001; in-hospital major morbidity: AUC 0.73, *p* < 0.001; 30-day major morbidity: AUC 0.70, *p* < 0.001). POSSUM PS with a cutoff of 25 (defined in our study as a ‘Chole-POSSUM’ score) was then validated in a separate cohort of patients. It showed a 100% sensitivity and a 100% negative predictive value for mortality and a 96–97% negative predictive value for major complications.

**Conclusions:**

The Chole-risk score was externally validated, but the CHOLE-POSSUM stands as a more accurate prediction model. CHOLE-POSSUM is a reliable tool to stratify patients with ACC into a low-risk group that may represent a safe EC candidate, and a high-risk group, where new minimally invasive endoscopic techniques may find the most useful field of action.

*Trial Registration*: ClinicalTrial.gov NCT04995380.

**Supplementary Information:**

The online version contains supplementary material available at 10.1186/s13017-023-00488-6.

## Background

The prevalence of gallstones in the general population is 10–15%, and 20–40% of these patients will likely develop gallstone-related complications [1]. Acute calculous cholecystitis (ACC) represents the first clinical presentation in 10–15% of patients with gallstone-related complications [1]. The most used guidelines for managing ACC are the Tokyo guidelines (TG) [2–4] and the World Society of Emergency Surgery (WSES) guidelines (GL) [1, 5]. TG and WSES GL agree to identify early cholecystectomy (EC) as the first-line therapy for ACC. However, many controversies exist about the contraindications of EC and the selection of patients at high risk when a surgical approach is performed.

According to the TG [6], patients with a contraindication for EC should be selected using ACC grade associated with Charlson Comorbidity Index (CCI), American Society of Anesthesiologists—Performance Status (ASA-PS), and the presence of organ dysfunctions. However, WSES GL identify as the only real contraindication for EC patients who refuse or are not suitable for surgery, but the characteristics of this category are not well defined. Recent data showed that there may be viable alternatives that are less invasive than EC for treating ACC in high-risk patients, e.g., transmural ultrasound-guided gallbladder drainage (TUGD) with lumen-apposing self-expandable metal stents (LAMSs) [7]. Still, we lack a reliable tool to select the group of patients who could benefit the most from these non-surgical procedures.

In 2021, Di Martino et al. [8] created a relatively simple and easily reproducible score (the Chole-risk score) to select patients with a higher risk of complicated course after EC for ACC. The model was validated by an internal retrospective analysis. Recently, some other well-known risk prediction models (POSSUM [9, 10], modified Frailty Index (mFI) [11], CCI [12]) have been applied and validated for EC in patients with ACC, but almost all are missing a formal perspective or external validation. A systematic review and meta-analysis looking at the ability of prognostic factors or risk prediction models to predict outcomes in patients with ACC after EC, showed that, up to now, no reliable model has been identified [13]. The only available comparison of three risk assessment scores (ASA-PS, APACHE II, and POSSUM) highlighted a significant association of the three scores with morbidity and mortality and the APACHE II seems to be the best risk predictor. Nevertheless, it is still limited to patients with perforated cholecystitis [14]. Additionally, to perform an APACHE II score, an ABG is necessary and it is a laboratory test not routinely performed around the world. For these reasons, the WSES GL do not suggest the use of any prognostic model in patients with ACC [1].

In this context, the validation and comparison of Scores for Prediction of RIsk for postoperative major Morbidity after cholecystectomy in Acute Calculous Cholecystitis (S.P.Ri.M.A.C.C.) study was conceived as a prospective multicenter observational study on patients with ACC candidate to EC. It aims to prospectively validate the Chole-risk score in predicting postoperative complications in patients undergoing EC for ACC compared with other preoperative risk prediction models (the POSSUM Physiological Score (PS), the mFI, the CCI, the ASA-PS, the APACHE II score, and the severity grade of ACC according to TG).

## Methods

### Ethical considerations

The study protocol was approved by the medical Ethics Board of the trial coordinating center at the IRCCS San Matteo Hospital, Pavia (Italy). Secondary approvals were obtained from all local ethics committees in the participating centers. Patients gave orally and written informed consent prior to inclusion. The SPRIMACC trial was conducted in accordance with the declaration of Helsinki.

### Design

The S.P.Ri.M.A.C.C. study is a WSES prospective multicenter observational study. From 1st September 2021 to 1st September 2022, 1,253 patients from 79 centers located in 19 different countries were included in the study. It was registered in ClicalTrial.gov with the following identifier: NCT04995380 and adhered to TRIPOD guidelines/methodology [15]. Patients were recruited in the preoperative period by surgeon investigators of the centers who joined the study after the examination of the patient and instrumental and biochemical investigations that allowed them to diagnose ACC.

### The Chole-risk score

The Chole-risk Score was developed using four groups of preoperative variables: (a) previous abdominal surgery or previous percutaneous cholecystostomy; (b) patient comorbidities such as diabetes and CCI > 6; (c) predictors of concomitant bile duct stones such as increased total bilirubin > 2 mg/dL and dilated bile duct; (d) predictors of difficult cholecystectomy such as perforated gallbladder and severity grade (1 vs 2–3 according to 2018 TG).

Each group can score either 0 or 1 for a positive variable. The score with its risk assessment was made available online at https://www.calconic.com/calculator-widgets/cholerisk/5f00380606e42a00296f59de?layouts=true.

### Study variables

The primary endpoint of S.P.Ri.M.A.C.C. study was the composite outcome already used in the work by Di Martino et al. [8], including 30-day postoperative major morbidity (intended as Clavien-Dindo ≥ 3a complications), length of stay (LOS) > 10 days and readmission within 30 days from the discharge. The secondary endpoint of the study was to prospectively validate and compare the performance of preoperative risk prediction models (the POSSUM Physiological Score (PS), the mFI, the CCI, the ASA-PS, the APACHE II score, the severity grade of ACC according to TG) in predicting in-hospital mortality, 30-day mortality, in-hospital major morbidity (intended as Clavien-Dindo ≥ 3a complications) and 30-day major morbidity in patients with ACC undergoing EC.

### Inclusion and exclusion criteria

Inclusion criteria were (1) have a diagnosis of ACC as defined by 2018 TG criteria, (2) be a candidate for EC during the index admission (other surgical techniques, e.g. open or bailout procedures such as subtotal cholecystectomy, were not reasons for intraoperative exclusion), (3) be ≥ 18 years old, (4) be stratified for the risk of common bile duct stones, and, in case of confirmation, receive preoperative ERCP, (5) provide a signed and dated informed consent form and (6) be willing to comply with all study procedures and be available for the duration of the study.

Exclusion criteria were (1) pregnancy or lactation, (2) acute cholecystitis not related to a gallstone etiology, (3) onset of symptoms > 10 days before cholecystectomy (patients with ACC associated with common bile duct stones who underwent preoperative ERCP could have been included if they had received EC within 10 days from onset of symptoms), (4) concomitant cholangitis or pancreatitis, (5) intraoperative treatment of common bile duct stones, or (6) anything that would increase the risk for the patient or preclude the individual’s full compliance with or completion of the study.

The Chole-risk score considers the presence of predicting factors for concomitant common bile duct stones as a risk factor for a complicated postoperative outcome after EC for ACC. However, as stated in the inclusion criteria, all the included patients with common bile duct stones have received a preoperative ERCP.

### Statistical analysis

*Sample size:* Sample size to validate the diagnostic performance of the Chole-risk score was calculated with the aim to obtain a minimum of 100 events and 100 nonevents [15–17]. Considering an incidence of 15.1% of the composite outcome in the study by Di Martino’s trial [8], the number of patients needed to reach 100 events and 563 nonevents was 663 enrolled patients. The time to complete enrollment was fixed at one year, and the follow-up at 30 days from discharge. Patients with missing data were excluded from the analysis.

*Statistical comparison and prediction models:* The Chi-square test was used to compare categorical data. A Cochran-Armitage test of trend was run to determine whether a linear trend existed between the Chole-risk score and the composite outcome. A two-tailed *p* < 0.05 was considered statistically significant. To assess the prediction accuracy of the analyzed prediction models, receiver operating characteristic (ROC) curves were generated for each scoring system. The area under the ROC curve (AUC) was used to compare the diagnostic abilities of the scoring systems. The study population was divided into a “derivation cohort” and a “validation cohort”, made of patients with a ratio of 1:1, and Youden’s index was adopted to find the best cutoff value in the derivation cohort. The identified cutoff was then assessed in the validation cohort and the accuracy of each cutoff was identified. Then, we identified a common cutoff for in-hospital mortality, 30-day mortality, in-hospital major morbidity and 30-day major morbidity, favoring sensitivity.

## Results

**Fig. 1:**
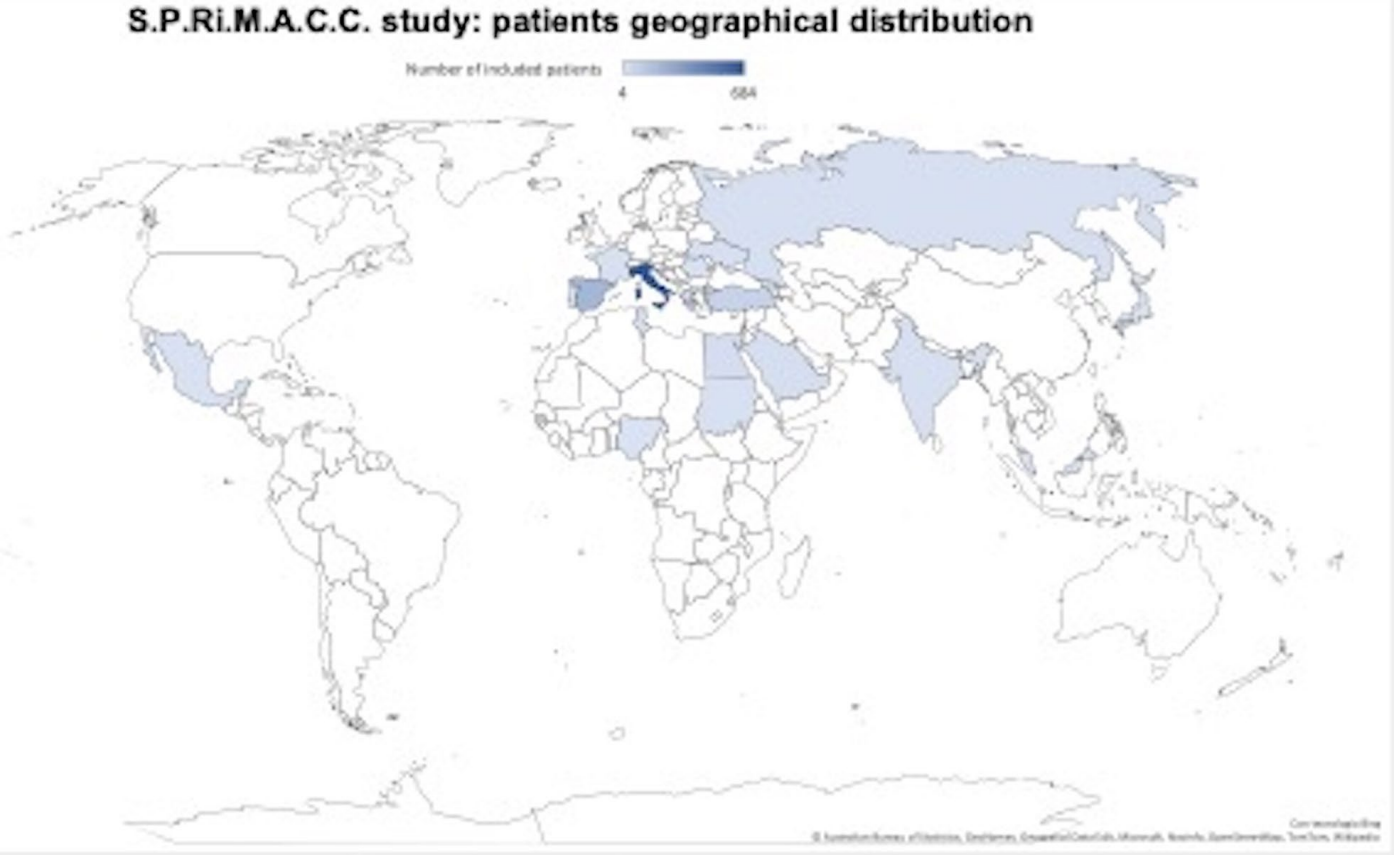
Patients geographical distribution according to S.P.Ri.M.A.C.C. participants

A total of 1429 consecutive patients were enrolled from 1st September 2021 to 1st September 2022. After excluding 176 patients for missing data, 1,253 patients from 79 centers located in 19 different countries were included (Additional file 1: Centers included in S.P.Ri.M.A.C.C. study with number of patients; Fig. 1). The patients’ preoperative characteristics and scores are shown in Table 1. The in-hospital major morbidity rate (intended as Clavien-Dindo ≥ 3a complications) was 5.2%, the 30-day major morbidity rate 6.6%, the in-hospital mortality rate was 1.0% and the 30-day mortality rate was 1.1%. The rate of positive Chole-risk outcome (30-day postoperative major morbidity or LOS > 10 days or 30-day readmission) was 14.3%. The intraoperative and postoperative outcomes are reported in Table 2.

**Table 1:** Patients’ characteristics

SCORE	% (N = 1253) mean ± SD (min–max)
ACC grade	
1	32.2
2	67.3
3	0.5
ASA	
1	21.1
2	42.2
3	25.5
4	3.8
5	0.2
Chole-risk	
0	18.0
1	44.4
2	25.9
3	7.4
4	0.8
M-FI	1.0 ± 1.4 (0–8)
Charlson comorbidity index	2.5 ± 2.4 (0–19)
POSSUM (Physiological Score)	20.8 ± 6.5 (12–59)
APACHE II	6.6 ± 5.1 (0–71)
Age	59.4 ± 17.0 (18–98)

ACC, Acute Calcolous Cholecystitis; ASA, American Society of Anesthesiologists—Performance Status; M-FI, modified Frailty Index

**Table 2:** Patients’ postoperative outcomes

	% (N = 1253) mean ± SD (min–max)
In-hospital major complication rate	5.2
30-day major complication rate	6.6
In-hospital mortality rate	1.0
30-day mortality rate	1.1
Chole-risk outcome	14.2
Laparotomic EC	7.5
Conversion rate	8.4
Bail out procedures	8.6
Intraoperative complication rate	4.1
Intraoperative mortality rate	0.0
Length of stay	5.5 ± 5.5 (1–55)

### Validation of Chole-risk

18.0% of included patients had a Chole-risk score of 0, 44.4% of 1, 25.9% of 2, 7.4% of 3 and 0.8% of 4. The Cochran-Armitage test of trend showed a statistically significant linear trend (*p* < 0.001) with a higher Chole-risk score associated with a higher proportion of patients with the composite outcome (Table 3).

**Table 3:** Chole-risk validation (*p* < 0.001)

Chole-risk	Complicated course* %
0	7.6
1	13.2
2	17.0
3	29.0
4	50.0

*30-day postoperative major morbidity or LOS > 10 days or 30-day readmission

### Comparison of risk prediction models

**Fig. 2:**
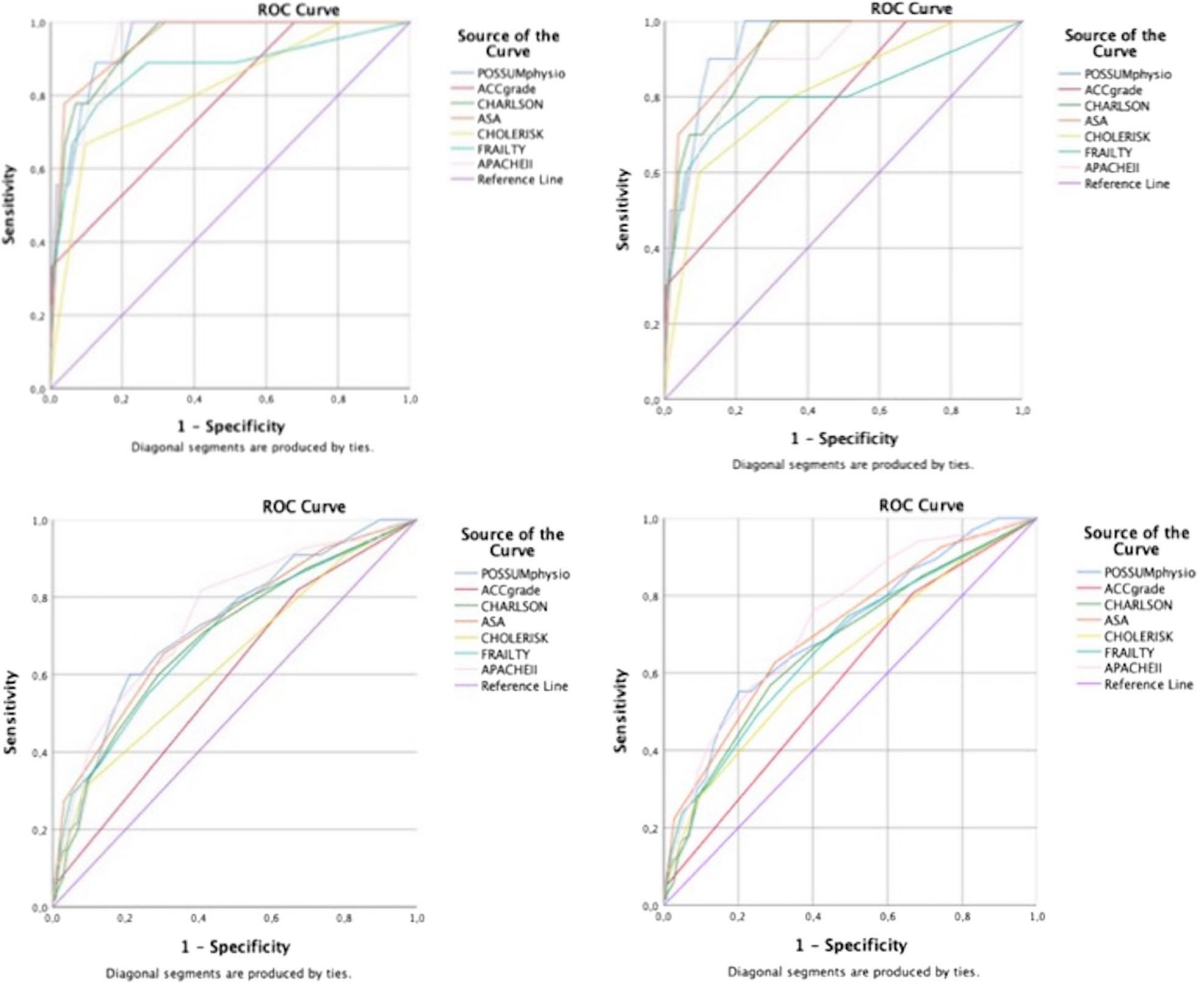
ROC curves of POSSUM physiological score, Acute Calcolous Cholecystitis (ACC) severity grade (according to the 2018 Tokyo Guidelines), Charlson Comorbidity index, ASA-PS, Chole-risk, modified Frailty Index, APACHE II for in-hospital mortality (**a**), 30-day mortality (**b**), in-hospital major morbidity (**c**), 30-day major morbidity (**d**) in patients with ACC after EC

Figure 2 and Table 4 report the ROC curves and AUCs of the tested score for each outcome.

- In-hospital mortality
  The three risk prediction models that best predicted in-hospital mortality were ASA-PS (AUC 0.946, *p* < 0.001), POSSUM PS (AUC 0.944, *p* < 0.001) and APACHE II (AUC 0.942, *p* = 0.023).
- 30-day mortality
  The three scores that best predicted 30-day mortality were POSSUM PS (AUC 0.941, *p* < 0.001), ASA-PS (AUC 0.934, *p* < 0.001) and CCI (AUC 0.922, *p* < 0.001).
- In-hospital major morbidity
  The three models that best predicted in-hospital major morbidity were APACHE II (AUC 0.749, *p* < 0.001), POSSUM PS (AUC 0.731, *p* < 0.001) and ASA-PS (AUC 0.724, *p* < 0.001).
- 30-day major morbidity
  The three models that best predicted 30-day major complications were APACHE II (AUC 0.735, *p* < 0.001), ASA-PS (AUC 0.710, *p* < 0.001), POSSUM PS (AUC 0.703, *p* < 0.001).

**Table 4:** Areas under the curves for in-hospital mortality, 30-day mortality, in-hospital major morbidity, 30-day major morbidity in patients with acute calculous cholecystitis after early cholecystectomy

Outcome	Risk prediction models	AUC	Std. Error	*p* value	95% Confidence interval	
					Lower bound	Upper bound
In-hospital mortality	POSSUM physiological score	0.944	0.023	< 0.001	0.898	0.989
	ACC grade	0.772	0.076	0.005	0.624	0.920
	Charlson Comorbidity Index	0.940	0.027	< 0.001	0.887	0.993
	ASA	0.946	0.026	< 0.001	0.894	0.998
	Chole-risk	0.815	0.077	0.001	0.663	0.967
	m-Frailty	0.870	0.078	< 0.001	0.716	1.000
	APACHEII	0.942	0.023	< 0.001	0.897	0.986
30-day mortality	POSSUM physiological score	0.941	0.021	< 0.001	0.899	0.982
	ACC grade	0.762	0.071	0.004	0.622	0.902
	Charlson Comorbidity Index	0.922	0.030	< 0.001	0.862	0.981
	ASA	0.934	0.028	< 0.001	0.879	0.989
	Chole-risk	0.811	0.070	0.001	0.673	0.948
	m-Frailty	0.808	0.093	0.001	0.625	0.991
	APACHEII	0.900	0.045	< 0.001	0.813	0.988
In-hospital major morbidity	POSSUM physiological score	0.731	.035	< 0.001	0.662	0.800
	ACC grade	0.589	0.039	0.026	0.514	0.665
	Charlson Comorbidity Index	0.694	0.038	< 0.001	0.619	0.769
	ASA	0.724	0.037	< 0.001	0.651	0.798
	Chole-risk	0.638	0.041	0.001	0.557	0.719
	m-Frailty	0.699	0.039	< 0.001	0.623	0.775
	APACHEII	0.749	0.035	< 0.001	0.681	0.817
30-day major morbidity	POSSUM physiological score	0.703	0.034	< 0.001	0.637	0.770
	ACC grade	0.584	0.035	0.023	0.515	0.653
	Charlson Comorbidity Index	0.670	0.036	< 0.001	0.600	0.741
	ASA	0.710	0.034	< 0.001	0.644	0.777
	Chole-risk	0.638	0.037	< 0.001	0.564	0.711
	m-Frailty	0.671	0.036	< 0.001	0.600	0.742
	APACHEII	0.735	0.031	< 0.001	0.674	0.796

The two models that fall into the three best scores for all secondary outcomes were the ASA-PS and the POSSUM PS.

### Cutoff derivation

**Fig. 3:**
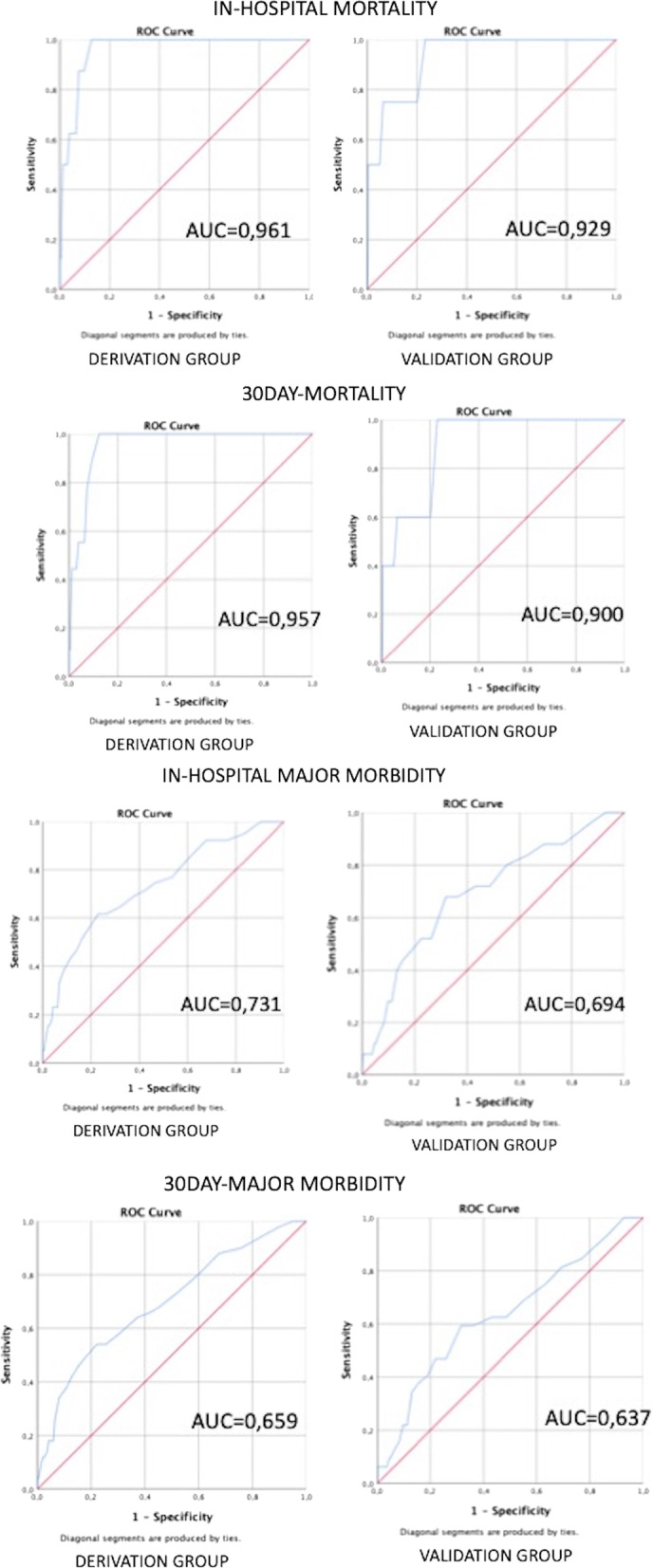
ROC curves of POSSUM Physiological Score of the derivation and validation groups for in-hospital mortality (**a**), 30-day mortality (**b**), in-hospital major morbidity (**c**), 30-day major morbidity (**d**)in patients with ACC after EC

The derivation group for the cutoff establishment for POSSUM PS was made of patients from 1 to 624, while the validation group of patients from 625 to 1253. The ROC curves and AUCs of POSSUM PS in the **derivation group** for in-hospital mortality, 30-day mortality, in-hospital major morbidity and 30-day major morbidity are shown in Fig. 3. The best common cutoff of POSSUM PS for the outcomes in the derivation group was 25 (< 25 vs ≥ 25).

We called the POSSUM PS with the tailored cutoff of 25 for selecting high-risk patients with ACC candidate to EC, CHOLE-POSSUM score.

### Cutoff internal validation

In the derivation group, 25.3% of patients had a POSSUM PS ≥ 25. Compared to patients with POSSUM PS < 25, patients with POSSUM PS ≥ 25 had significant higher in-hospital mortality (5.4% vs 0%, *p* < 0.001), 30-day mortality (6.2% vs 0%, *p* < 0.001), in-hospital major morbidity (16.3% vs 3.5%, *p* < 0.001), 30-day major morbidity (19.0% vs 5.3%, *p* < 0.001) (as shown in Table 5). The sensitivity, specificity, positive predictive value and negative predictive value of the CHOLE-POSSUM score in the derivation group were in sequence as follows: 100%, 76%, 5% and 100% for in-hospital mortality and for 30-day mortality; 62%, 77%, 16% and 97% for in-hospital major morbidity; 54%, 78%, 19% and 95% for 30-day major morbidity.

**Table 5:** Internal (derivation group) and external (validation group) validation of 25 as POSSUM Physiological Score cutoff to select high-risk patients with acute calculous cholecystitis for early cholecystectomy

Outcome	Group	Derivation group (%)	*p* value	Validation group (%)	*p* value
In-hospital mortality	POSSUM PS < 25	0	< 0.001	0	0.003
	POSSUM PS > = 25	5		3	
30-day mortality	POSSUM PS < 25	0	< 0.001	0	0.001
	POSSUM PS > = 25	6		4	
In-hospital major morbidity	POSSUM PS < 25	4	< 0.001	3	0.002
	POSSUM PS > = 25	16		10	
30-day major morbidity	POSSUM PS < 25	5	< 0.001	4	0.002
	POSSUM PS > = 25	19		11	

### Cutoff external validation

The ROC curves and AUCs of POSSUM PS in the **validation group** for in-hospital mortality, 30-day mortality, in-hospital major morbidity and 30-day major morbidity are reported in Fig. 3.

In the validation group, 23.4% of patients had a POSSUM PS ≥ 25. Compared to patients with POSSUM PS < 25, patients with POSSUM PS ≥ 25 had a significantly higher in-hospital mortality (2.9% vs 0.0%, *p* = 0.003), 30-day mortality (3.7% vs 0.0%, *p* = 0.001), in-hospital major morbidity (9.5% vs 2.7%, *p* = 0.002), 30-day major morbidity (11.2% vs 3.8%, *p* = 0.002) (Table 5). The sensitivity, specificity, positive predictive value and negative predictive value of the CHOLE-POSSUM score in the validation group was in sequence as follows 100%, 77%, 3% and 100% for in-hospital mortality; 100%, 77%, 4% 100% for 30-day mortality; 52%, 76%, 10% and 97% for in-hospital major morbidity; 47%, 78%, 11% and 96% for 30-day major morbidity.

## Discussion

Up to now, evidence in the field of ACC has defined EC as the gold standard of treatment, also in high-risk patients. A recent randomized controlled trial (CHOCOLATE) [18] compared EC and percutaneous gallbladder drainage (PTGBD) in high-risk patients (APACHE II score ≥ 7) with ACC and showed a higher major complication rate, a higher reintervention rate and a higher rate of recurrent biliary disease in PTGBD. In light of these data, WSES GL recommend EC also in high-risk patients. However, cholecystectomy in the setting of ACC is not a surgery without complications: significant data in the literature show mortality rates up to 3–4% for patients older than 80 years old [19] or with CCI > 5 [12], rate of 15.5% for patients with perforated gallbladder [14] and up to 46.3% for patients with ASA-PS III-IV [20]. In recent years, new non-surgical approaches—especially endoscopic procedures—emerged as alternative treatments for high-risk patients with ACC. Among these, according to the WSES GL, TUGD with LAMSs could be considered a safe, effective, and definitive alternative to PTGBD [1]. Actually, a recent randomized controlled trial (DRAC 1) [7] compared TUGD with PTGBD in high-risk patients (identified with one of the following: age ≥ 80, ASA-PS ≥ 3, age-adjusted CCI > 5 or Karnofsky score < 50) with ACC, and evidenced improved outcomes in TUGD group, as lower 1-year and 30-day adverse events, lower reintervention rate, lower rate of unplanned readmissions, lower rate of recurrent cholecystitis, lower pain and lower analgesic requirements. Some questions remain unanswered: which patients are suitable for these treatments and how they may be selected. To address these questions, some authors applied well-known preoperative risk prediction models to the setting of ACC [9–12, 21, 22], while other authors tried to create new tailored scores [8].

The WSES S.P.Ri.M.A.C.C. study aimed at clarifying which of these models are valid and reliable in such a setting.

First of all, the Chole-risk score has been prospectively validated, showing a good correlation with a complicated postoperative course. Then, the performance and the discrimination capacity of existing scores, including the Chole-risk, were compared to select the most reliable, applicable and valuable risk prediction model for a complicated postoperative course in these patients. The ideal score should be very sensitive, more than specific, in order not to miss high-risk patients. Furthermore, it should consider both preexisting patients’ comorbidity and the clinical conditions at the moment of EC. Finally, it should be simple to apply and should not require further tests than those performed in normal clinical practice (e.g. ABG).

The analyzed scores seem to predict mortality with high accuracy, while they showed, in general, lower performances in predicting major morbidity. Actually, in-hospital and 30-day mortality are the outcomes that a clinician would like to avoid the most when making a therapeutic decision on a patient with ACC suitable for EC. Furthermore, the scores that consider only patients’ conditions at the moment of EC and do not consider preexisting comorbidity (e.g. ACC grade derived from 2018 TG) reported the worst AUCs in our study.

Looking at the ROC curves, the two best models according to our analysis were the POSSUM PS and the ASA-PS: the ASA-PS includes only patient’s preexisting conditions and could be subjective, the POSSUM PS is an objective score and considers both patient’s comorbidity and patient’s conditions due to ACC. In light of this fact, POSSUM PS could be considered the best risk prediction model for a complicated course after EC for ACC.

The POSSUM score was proposed by Copeland et al. in 1991 [23] as a method for normalizing patient data, so that the direct comparison of patient outcomes could be made. It includes a PS, calculated in the preoperative time, and an Operative Severity Score (OS) calculated at the time of surgery. These scores are then inserted into two formulas [23], and risks of both mortality and morbidity can be predicted for the workload of each surgical team. The POSSUM score has been validated for hepato-biliary-pancreatic surgery [24, 25], gastric surgery [26], colorectal surgery [27] and emergency laparotomies [28]. The POSSUM score had already a validation in patients with ACC underwent EC or medical therapy [9, 10], but, up to now, a formal prospective validation of POSSUM for EC in patients with ACC was lacking. We considered the POSSUM PS, and not the OS, because the target of our study was a model that could be completely calculated in the preoperative period: according to this idea, a surgeon could be aware of high-risk patient predictive factors at the moment of clinical decisions.

The CHOLE-POSSUM could be defined as the POSSUM PS with a cutoff of 25, tailored to predict major morbidity and mortality in patients with ACC candidate to EC. This cutoff was internally and externally validated in the SPRIMACC population. The CHOLE-POSSUM has a 100% sensitivity and a 100% NPV in predicting mortality. Furthermore, it has a 96–97% NPV in predicting major complications. For these reasons, the CHOLE-POSSUM could be considered an excellent tool to select patients with ACC that can be safe candidates for EC without, ideally, a risk of postoperative mortality and with an acceptable risk of major complications. These “low-risk patients” represent about 75% of the population with ACC. On the other hand, patients with a CHOLE-POSSUM ≥ 25 have a risk of 30-day postoperative mortality at least four times higher than the general population, so, probably, for the latter less invasive therapeutical procedures (e.g., TUGD with LAMSs) should be considered.

Future trials should be designed to find the best treatment for ACC in the subgroup of high-risk patients. The CHOLE-POSSUM can select the patients who will constitute the study population for these trials. In this regard, the "Surgical vs Endoscopic Treatments as ImmunoModulating Interventions in High-Risk Acute Calculous Cholecystitis (SETIMIHRACC Study)" has recently been approved by the medical Ethics Board of the trial coordinating center at the IRCCS San Matteo Hospital, Pavia (Italy) and it will soon begin in Italy. In this trial, high-risk patients with ACC, selected using CHOLE-POSSUM, will be randomized to receive EC or TUGD with LAMSs.

There are some limits of the study. First, the sample size is tailored to the first outcome. However, there are no generally accepted approaches to estimate the sample size requirements for studies comparing the performance of risk prediction models, which is the secondary objective. Then, all available data on the database were used to maximize the power and generalizability of the results. Another limitation is that, while the CHOLE-POSSUM score has a high NPV, it shows a low PPV. This may be mainly related to the low pretest probability of mortality and morbidity in this group of patients. However, the test used must be able to identify as many patients at high risk as much as possible, to expose them as less as possible to the surgical risk, and also at the expense of specificity and PPV. Lastly, although EC is a worldwide standardized intervention, there is no geographical uniformity of the sample worldwide, with a prevalence of Italian and Spanish patients (as shown in Fig. 1).

## Conclusions

The Chole-risk score was externally validated, but the study has defined the best existing risk prediction model for a complicated course after EC in patients with ACC as the POSSUM PS, with the best cutoff to select high-risk patients to be 25. This allows us to stratify ACC patients into a low-risk group that can represent a safe EC candidate, and a high-risk group where new minimally invasive endoscopic techniques may be the most proper management choice. Moreover, the CHOLE-POSSUM can select the high-risk patients who will constitute the future study population for these techniques.

## Supplementary Information

**Additional file 1.** Centers included in S.P.Ri.M.A.C.C. study with number of patients.

## Data Availability

The datasets generated and/or analyzed during the current study are not publicly available but are available from the corresponding author on reasonable request.
