# Supplementary material for: Prediction of morbidity and mortality after early cholecystectomy for acute calculous cholecystitis: results of the S.P.Ri.M.A.C.C. study
Source: World J Emerg Surg. 2023 Mar 18;18:20. doi: 10.1186/s13017-023-00488-6 (PMC10024826; doi:10.1186/s13017-023-00488-6)
Supplement: Supplementary file 1 — Additional file 1. Centers included in S.P.Ri.M.A.C.C. study with number of patients. [file 13017_2023_488_MOESM1_ESM.docx]

Additional file 1. Centers included in S.P.Ri.M.A.C.C. study with number of patients

| Country | Centre | City | NUMBER OF INCLUDED PATIENTS |
| --- | --- | --- | --- |
| Croatia | Department of Surgery, University Hospital Centre Zagreb | Zagreb | 16 |
| Egypt | Department of General Surgery, Mansoura University Hospitals | Mansoura | 24 |
|  | University Faculty of Medicine | Tanta | 2 |
| France | CHU | Nice | 9 |
|  | Dpt of Emergency, Digestive and Metabolic Minimally invasive surgery, Poissy and Saint Germain en Laye Hospitals | Poissy | 5 |
| Georgia | State Medical University | Tbilisi | 17 |
| Greece | Hippocration General Hospital of Athens | Athens | 9 |
|  | National and Kapodistrian University of Athens, Laikon General Hospital | Athens | 5 |
|  | Saint Savvas Cancer Hospital | Athens | 3 |
|  | Naval and Veterans Hospital, Department of Surgery | Athens | 1 |
|  | University General Hospital Attikon-UoA | Chaidari | 4 |
|  | Surgery Department, General Hospital of Chania - Saint George | Crete | 4 |
|  | Surgery University Hospital of Heraklion, Medical School of Heraklion | Crete | 4 |
|  | General Hospital George Papanikolaou | Thessaloniki | 3 |
| India | Maulana Azad Medical College | New Delhi | 17 |
| Italy | Chirurgia generale, Ospedale Monsignor Galliano | Acqui Terme | 11 |
|  | Lorenzo Bonomo Hospital | Andria | 55 |
|  | University of Bari | Bari | 14 |
|  | Chirurgia 2, ASST Spedali Civili of Brescia | Brescia | 39 |
|  | Chirurgia 3, ASST Spedali Civili of Brescia | Brescia | 17 |
|  | University Hospital | Cagliari | 11 |
|  | PO Santissima Trinità | Cagliari | 31 |
|  | Azienda Policlinico Università di Catania | Catania | 8 |
|  | Bufalini Hospital | Cesena | 28 |
|  | UOC of General and Minimally Invasive Surgery, San Paolo Hospital | Civitavecchia | 36 |
|  | Azienda Ospedaliera Santa Croce e Carle | Cuneo | 6 |
|  | Azienda Ospedaliero-Universitaria di Ferrara, Arcispedale Sant'Anna | Ferrara | 4 |
|  | University Hospital | Foggia | 2 |
|  | Ospedale Morgagni Pierantoni | Forlì | 10 |
|  | Ospedale del Delta | Lagosanto | 10 |
|  | ASST Santi Paolo e Carlo | Milano | 6 |
|  | Chirurgia generale Trauma team, Ospedale Niguarda | Milano | 17 |
|  | General and Emergency Surgery, School of Medicine and Surgery, Milano-Bicocca University | Monza | 15 |
|  | Surgical oncology and digestive surgery, A.O.U. San Luigi Gonzaga | Orbassano | 10 |
|  | FBF Buccheri La Ferla | Palermo | 11 |
|  | Fondazione IRCCS Policlinico San Matteo | Pavia | 29 |
|  | AO Ospedali Riuniti Marche Nord | Pesaro | 34 |
|  | G, Da Saliceto Hospital | Piacenza | 7 |
|  | ASL2 Savonese | Pietra Ligure | 48 |
|  | Chirurgia generale Ospedale E. Agnelli | Pinerolo | 15 |
|  | Ospedale Immacolata Concezione | Piove di Sacco | 9 |
|  | "S.Stefano" New Hospital | Prato | 13 |
|  | Reggio Emilia, ASMN | Reggio Emilia | 8 |
|  | Ospedale di Rho - ASST Rhodense | Rho | 10 |
|  | Chirurgia Generale Ospedale degli Infermi | Rivoli | 20 |
|  | San Filippo Neri Hospital | Rome | 26 |
|  | Policlinico Umberto I | Rome | 26 |
|  | University Tor Vergata | Rome | 8 |
|  | UOC Chirurgia Bariatrica, Ospedale San Carlo di Nancy | Rome | 3 |
|  | University Hospital "S. Giovanni di Dio e Ruggi d'Aragona" | Salerno | 16 |
|  | Chirurgia Generale d'Urgenza e PS, AOU Città della Salute e della Scienza | Torino | 37 |
|  | General Surgery Department, Cattinara University Hospital, ASUGI | Trieste | 7 |
|  | Ospedale San Bortolo | Vicenza | 27 |
| Japan | Kurashiki Central Hospital | Kurashiki | 8 |
| Malaysia | Department of Surgery, Hospital Universiti Sains Malaysia | Kota Bharu | 10 |
| Mexico | Instituto Mexicano del seguro social | Ciudad de México | 4 |
|  | Regional Hospital of High Speciality of Bajio | Leon Guanajuato | 6 |
| Nigeria | Aminu Kano Teaching Hospital | Kano | 4 |
| Portugal | Centro Hospitalar Tondela-Viseu | Viseu | 14 |
| Romania | Emergency Hospital of Bucharest | Bucharest | 11 |
| Russia | Immanuel Kant Baltic Federal University, Regional Clinic Hospital | Kaliningrad | 20 |
| Saudi Arabia | General Surgery, King Saud Medical City | Riyadh | 25 |
| Spain | Department of Surgery and Liver Transplantation. Hospital General Universitario Dr. Balmis | Alicante | 9 |
|  | Hospital universitario Donostia | Donostia-San Sebastian | 15 |
|  | Hospital Universitario de La Princesa | Madrid | 69 |
|  | Hospital Universitario Príncipe de Asturias | Madrid | 31 |
|  | Emergency Surgery Unit, Parc Tauli Hospital | Sabadell | 10 |
|  | Urduliz Hospital | Urduliz | 58 |
|  | Department of Surgery, Miguel Servet University Hospital | Zaragoza | 25 |
| Sudan | Kuwaiti Specialized Hospital | Khartoum | 15 |
| Tunis | Bizerte hospital | Bizerte | 12 |
| Turkey | Sakarya Training and Research Hospital | Adapazari/Sakarya | 1 |
|  | Gazi University, School of Medicine, Department of Surgery | Ankara | 3 |
|  | Department of Gastroenterological Surgery, Dicle University School of Medicine | Diyarbakir | 5 |
|  | Kanuni Sultan Süleyman Training and Research Hospital | Instanbul | 1 |
|  | Bakirkoy Dr Sadi Konuk Training and Research Hospital | Instanbul | 2 |
|  | Tepecik Training and Research Hospital | Konak/İzmir | 64 |
|  | Samsun Training and Research Hospital | Samsun | 5 |
| Ukraine | Regional Clinical hospital | Lviv | 19 |
